# 69 Early Post-operative Mobilization After Treatment of Burn Wounds with Autologous Skin Cell Suspension

**DOI:** 10.1093/jbcr/irac012.072

**Published:** 2022-03-23

**Authors:** Jenna C Kelly, Emma C DeJournette, Steven A Kahn

**Affiliations:** South Carolina Burn Center at MUSC, Charleston, South Carolina; MUSC Hospital, Charleston, South Carolina; Medical University of South Carolina, Charleston, South Carolina

## Abstract

**Introduction:**

Early mobilization has become popular in the literature and the benefits in critically ill patients are well-documented. However, early mobilization in burn care is undefined. Clinical practice guidelines regarding post-operative mobilization (POM) of patients with burn injury, especially after autografting, are limited, resulting in significant practice variance among burn centers. Furthermore, data on mobilization after treatment with autologous skin cell suspension (ASCS), is even more limited, and wider practice variation exists. We hypothesize early POM is safe and does not compromise healing or lead to graft loss.

**Methods:**

A retrospective chart review was performed to examine mobility interventions utilized for patients, with mixed partial and full thickness burns, who received ASCS with polylactic acid sheet, with or without meshed splint-thickness skin grafting (STSG), over 6 months. Data included demographics, operative procedure, dressing, post-operative restrictions, POM date by burn therapy, and presence of graft loss ( >25% of grafted area with need for regrafting). The data was analyzed to corelate POM day and graft success. Initially, the authors utilized manufacturer splinting guidelines before becoming more judicious with aggressive early mobilization. Patients at risk of noncompliance and graft sheer were splinted.

**Results:**

Fifteen patients were included in the study. In those patients, 25 body areas were grafted with 17 areas receiving ASCS with polylactic acid sheet and 8 areas additionally receiving STSG. On post-operative day (POD) 0, four areas were splinted and no range of motion (ROM) was performed at the joint. The authors splinted 3 out of 4 patients (75%) due to initial manufacturer splinting guidelines. The 4th patient was splinted due to compliance concerns. For the other 21 areas, active ROM, functional mobility and performance of activities of daily living with functional movement was allowed on POD 0. A burn therapist actively treated 64% of patients on POD 0-1 and 100% of patients were treated by POD 2. Minimal patchy graft loss (median 1.5%) was noted in 6 grafted areas including 1 area which crossed a knee despite immobilization. No graft loss was related to POM and was attributed to factors including infection or wound conversion and, by definition, no significant graft loss was found.

**Conclusions:**

While the sample size is limited, this study suggests early POM of ASCS is safe and does not lead to graft loss. Considerations for mobilization techniques, such as ROM and functional mobility, should include joint involvement, presence of STSG with ASCS overspray, and other patient factors such as health literacy and compliance. Additional prospective studies should be dedicated to the examination of POM to develop clinical practice guidelines which can be widely utilized across burn therapy.

